# Association of Race and Ethnicity With Comorbidities and Survival Among Patients With COVID-19 at an Urban Medical Center in New York

**DOI:** 10.1001/jamanetworkopen.2020.19795

**Published:** 2020-09-25

**Authors:** Rafi Kabarriti, N. Patrik Brodin, Maxim I. Maron, Chandan Guha, Shalom Kalnicki, Madhur K. Garg, Andrew D. Racine

**Affiliations:** 1 Department of Radiation Oncology, Montefiore Medical Center and Albert Einstein College of Medicine, Bronx, New York; 2 Department of Pediatrics, Montefiore Medical Center and Albert Einstein College of Medicine, Bronx, New York

## Abstract

**Importance:**

As of May 11, 2020, there have been more than 290 000 deaths worldwide from severe acute respiratory syndrome coronavirus 2 (SARS-CoV-2), which causes coronavirus disease 2019 (COVID-19). Risk-adjusted differences in outcomes among patients of differing ethnicity and race categories are not well characterized.

**Objectives:**

To investigate whether presenting comorbidities in patients with COVID-19 in New York City differed by race/ethnicity and whether case fatality rates varied among different ethnic and racial groups, controlling for presenting comorbidities and other risk factors.

**Design, Setting, and Participants:**

This cohort study included 5902 patients who presented for care to the Montefiore Medical Center, a large urban academic medical center in the Bronx, New York, between March 14 and April 15, 2020, and tested positive for SARS-CoV-2 on reverse transcription quantitative polymerase chain reaction assay. Final data collection was April 27, 2020.

**Exposures:**

Patient characteristics, including self-identified ethnicity/race, age, sex, socioeconomic status, and medical comorbidities, were tabulated.

**Main Outcomes and Measures:**

Overall survival. Associations between patient demographic characteristics, comorbidities, and race/ethnicity were examined using χ^2^ tests, and the association with survival was assessed using univariable and multivariable Cox proportional hazards regression, based on time from positive COVID-19 test.

**Results:**

Of 9268 patients who were tested, 5902 ethnically diverse patients (63.7%) had SARS-CoV-2. Of these, 3129 patients (53.0%) were women, and the median (interquartile range) age was 58 (44-71) years. A total of 918 patients (15.5%) died within the study time frame. Overall, 1905 patients (32.3%) identified as Hispanic; 1935 (32.8%), non-Hispanic Black; 509 (8.6%), non-Hispanic White; and 171 (2.9%), Asian; the death rates were 16.2% (309), 17.2% (333), 20.0% (102), and 17.0% (29), respectively (*P* = .25). Hispanic and non-Hispanic Black patients had a higher proportion of more than 2 medical comorbidities with 654 (34.3%) and 764 (39.5%), respectively, compared with 147 (28.9%) among non-Hispanic White patients (*P* < .001). Hispanic and non-Hispanic Black patients were also more likely to test positive for COVID-19 than White patients, with 1905 of 2919 Hispanic patients (65.3%), 1935 of 2823 non-Hispanic Black patients (68.5%), and 509 of 960 non-Hispanic White patients (53.0%) having positive test results for SARS-CoV-2 (*P* < .001). While controlling for age, sex, socioeconomic status and comorbidities, patients identifying as Hispanic (hazard ratio, 0.77; 95% CI, 0.61-0.98; *P* = .03) or non-Hispanic Black (hazard ratio, 0.69; 95% CI, 0.55-0.87; *P* = .002) had slightly improved survival compared with non-Hispanic White patients.

**Conclusions and Relevance:**

In this cohort study of patients with COVID-19 who presented for care at the same urban medical center, non-Hispanic Black and Hispanic patients did not experience worse risk-adjusted outcomes compared with their White counterparts. This finding is important for understanding the observed population differences in mortality by race/ethnicity reported elsewhere.

## Key Points

**Question:**

Does the presentation of comorbidities in patients with coronavirus disease 2019 (COVID-19) differ by race/ethnicity, and is there a difference in case fatality rates among ethnic and racial groups when controlling for key risk factors?

**Findings:**

In a cohort study of 5902 patients with positive COVID-19 diagnosis treated at a single academic medical center in New York, non-Hispanic Black and Hispanic patients had a higher proportion of more than 2 medical comorbidities and were more likely to test positive for COVID-19 compared with their non-Hispanic White counterparts. However, their survival outcomes were at least as good as those of their non-Hispanic White counterparts when controlling for age, sex, and comorbidities.

**Meaning:**

In this study, non-Hispanic Black and Hispanic patients experienced similar outcomes as their non-Hispanic White counterparts after COVID-19 infection; this is critical to further understanding the observed population differences in mortality by race/ethnicity reported elsewhere.

## Teaser

This cohort study investigates whether presenting comorbidities in patients with coronavirus disease (COVID-19) in New York City differed by race/ethnicity and whether case fatality rates varied among different ethnic and racial groups controlling for presenting comorbidities and other risk factors.

## Introduction

As of May 11, 2020, there had been more than 4.1 million cases worldwide of severe acute respiratory syndrome coronavirus 2 (SARS-CoV-2), which causes coronavirus disease 2019 (COVID-19), with 290 000 deaths.^1^ In the United States, according to the Centers for Disease Control and Prevention (CDC), accessed on May 11, 2020, more than 1 324 000 cases had been identified, with more than 79 000 deaths recorded.^2^ Published reports are beginning to emerge characterizing the presentation of patients cared for in large health systems in New York and California.^3,4^ Despite this emerging evidence, a May 2020 article^5^ highlighted the lack of detailed information regarding ethnicity in reports on COVID-19 cases and outcomes.

The CDC recently reported on a sample of 1482 patients with confirmed COVID-19 who were hospitalized from March 1 to 30, 2020, with race/ethnicity information for 580 of them.^6^ They found that African Americans made up 33% of the sample of hospitalized patients while they represented 18% of the catchment population. By contrast, non-Hispanic White patients made up 45% of the hospitalized sample but 59% of the residents in the sampled area.^7^

These and other observations have led to much speculation regarding why patients from minority ethnic and racial groups appear to be particularly susceptible to infection with and complications from this new pathogen. Factors such as living conditions, including housing density and residential segregation; working conditions, especially acknowledging the overrepresentation of these populations among essential workers; the prevalence of underlying health conditions; and access to quality health care may affect this perceived association. As the number of patients with COVID-19 rapidly increases, understanding how ethnicity and race play into the outcomes of this disease is crucial. Health systems located in New York City are uniquely positioned to contribute to this evolving portrait because of the high volume of cases and the racial and ethnic diversity of the populations in this largest of US metropolitan areas. In particular, by examining the experience of an ethnically diverse sample of patients all cared for at a single institution, we can hold constant the associations of care received with outcomes and focus on whether race/ethnicity are independently associated with outcomes of patients with COVID-19.

Here we present a retrospective analysis of patients with COVID-19 who presented to a large urban medical center serving an ethnically and racially diverse population. The overall aim of the study was to characterize the presentation of patients with laboratory confirmed COVID-19 infections cared for at the Montefiore Medical Center located in the Bronx, New York, especially regarding differences among ethnic and racial groups. Because age is known to be the most potent factor associated with outcomes for COVID-19 infections, we posed three questions, as follows: (1) did the presentation of comorbidities differ by race/ethnicity, (2) controlling for age and comorbidities, did case fatality rates differ by race/ethnicity, and (3) did the association between comorbidities and survival vary by race/ethnicity?

## Methods

### Data Collection

In this cohort study, we used the electronic medical record to identify all patients who were tested for COVID-19 between March 14 and April 15, 2020, and presented for care to the Montefiore Medical Center, a large academic medical center located in the Bronx, New York, whether or not they were admitted as inpatients. SARS-CoV-2 positive status was determined based on reverse transcription quantitative polymerase chain reaction assay. Patients who were tested multiple times were categorized as positive if any of their tests indicated positivity. Patient characteristics, race/ethnicity, socioeconomic status (SES), comorbidities, and clinical outcomes were tabulated for patients who tested positive for COVID-19, with final data collection on April 27, 2020. Individual comorbidities included in the Charlson Comorbidity Index were collected as well as body mass index (BMI; calculated as weight in kilograms divided by height in meters squared) and hypertension. Comorbidities were included based on either *International Classification of Diseases, Ninth Revision *(*ICD*-*9*) or *International Statistical Classification of Diseases and Related Health Problems, Tenth Revision *(*ICD*-*10*) classification. Race/ethnicity were based on patient self-identification and categorized as Hispanic, non-Hispanic Black, non-Hispanic White, Asian, other (comprising non-Hispanic patients indicating their race as multiple selected, American Indian or Alaska Native, other, or some other race) or unknown/declined. SES was calculated by a representative summary score based on home address, using neighborhood information taking into account median household income, median value of housing units, occupation and education of inhabitants, and percentage of households receiving interest or net rental income.^8^ The SES score is a relative measure and should be interpreted as such, in which a lower SES score reflects lower socioeconomic status.

This study was approved by the institutional review board of the Albert Einstein College of Medicine, and informed consent was waived because this was a retrospective cohort study and because patient data were deidentified. The study followed the Strengthening the Reporting of Observational Studies in Epidemiology (STROBE) reporting guideline for cohort studies.

### Statistical Analysis

Associations between categorical variables of patient demographic characteristics, comorbidities, and race/ethnicity were examined using χ^2^ tests, and associations with continuous variables were assessed using 1-way analysis of variance tests. The associations between survival and patient demographic characteristics, comorbidities, ethnic and racial group, age, and SES were assessed using univariable and multivariable Cox proportional hazards regression, based on time from positive COVID-19 test, with patients still alive censored at time of last contact with our hospital system. All deaths occurring within the hospital were included, and any deaths occurring outside of the hospital were included from the in-house analytical software, which links continuously to the Social Security Administration mortality registry. The proportional hazards assumptions were examined via visual inspection of the Schoenfeld residuals for each model covariate.

Because 835 patients (14.1%) were missing information for BMI and 676 (11.5%) were missing SES scores, this could produce biased or inefficient results when analyzing only patients with complete information for all covariates. Therefore, missing values of BMI were imputed based on sex and age, and missing values of SES were imputed using sex, age, and area code. Imputations were performed using ordered logistic regression for BMI categories and SES quartiles, using 20 multiple imputation data sets.

Subgroup analyses of patients with known ethnicity identification were performed to compare the proportion of medical comorbidities among racial and ethnic groups in patients who were admitted to the hospital or those admitted to the intensive care unit (ICU). Stratified multivariable Cox regression analyses were performed to examine whether risk factors for death were different within each race/ethnicity strata. All statistical analyses were performed using Stata version 14.2 (StataCorp). Statistical significance was set at *P* < .05, and all tests were 2-tailed.

## Results

A total of 9268 patients who received care at Montefiore Medical Center were tested for COVID-19 between March 14 and April 15, 2020 (Figure), with Hispanic and non-Hispanic Black patients being more likely to test positive for COVID-19 (1905 of 2919 [65.3%] and 1935 of 2823 [68.5%], respectively vs 509 of 960 [53.0%] for non-Hispanic White patients; *P* < .001). We are reporting the outcomes of 5902 patients (63.7%) who tested positive at any time in the study period. The median (interquartile range) age was 58 (44-71) years, and 3129 patients (53.0%) were women. Overall, 1905 patients (32.3%) identified as Hispanic; 1935 (32.8%), non-Hispanic Black; 509 (8.6%), non-Hispanic White; 171 (2.9%), Asian; 792 (13.4%), other; and 590 (10.0%) had unknown race/ethnicity or declined to answer (Table 1). Patients identifying as non-Hispanic Black or Hispanic presented with a higher number of medical comorbidities (>2 comorbidities, 764 [39.5%] and 654 [34.3%]) and lower SES (lowest, second, third, and highest quartile: −4.08, −1.63, 0.91, and 1.68, respectively, and −6.39, −3.41, −1.66, and 1.33, respectively) compared with non-Hispanic White patients (>2 comorbidities, 147 [28.9%]; lowest, second, third, and highest SES quartile: −2.05, −0.68, 0.82, and 3.14, respectively). Conversely, slightly more non-Hispanic White patients were older than 80 years compared with non-Hispanic Black and Hispanic patients (96 [18.8%] vs 206 [10.6%] and 185 [9.7%]). Overall, 3912 of 5067 patients (77.2%) with complete comorbidity information had at least 1 medical comorbidity, and 2842 (56.1%) had at least 2. A total of 1942 of all 5902 patients (32.9%) had diabetes, and 2641 (44.7%) had hypertension.

**Figure.**
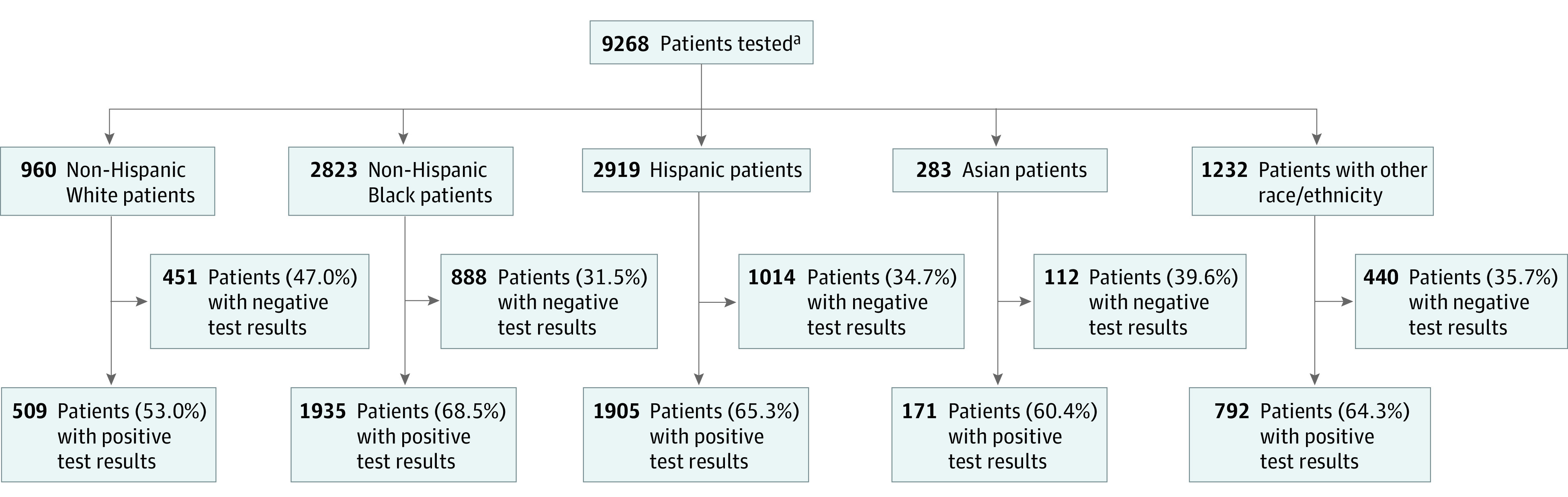
Positive and Negative Testing Rate for All Patients, by Ethnic and Racial Group ^a^Race/ethnicity data were available for 8217 individuals. For the 1051 patients whose race/ethnicity was unknown or who declined to answer, 590 (56.1%) had positive test results.

**Table 1.** Patient Demographic Characteristics, Stratified by Ethnicity and Race

Characteristic	Patients, No. (%)						*P* value
	All (N = 5902)	Non-Hispanic White (n = 509)	Non-Hispanic Black (n = 1935)	Hispanic (n = 1905)	Asian (n = 171)	Other or unknown race/ethnicity (n = 1382)	
Sex							
Men	2768 (46.9)	250 (49.1)	814 (42.1)	947 (49.7)	81 (47.4)	676 (48.9)	<.001
Women	3129 (53.0)	258 (50.7)	1121 (57.9)	956 (50.2)	90 (52.6)	704 (50.9)	
Age, y							
≤40	1255 (21.3)	72 (14.1)	303 (15.7)	436 (22.9)	38 (22.2)	406 (29.4)	<.001
41-60	2026 (34.3)	137 (26.9)	695 (35.9)	677 (35.5)	65 (38.0)	452 (32.7)	
61-80	2002 (33.9)	204 (40.1)	731 (37.8)	607 (31.9)	53 (31.0)	407 (29.5)	
>80	619 (10.5)	96 (18.9)	206 (10.6)	185 (9.7)	15 (8.8)	117 (8.5)	
Socioeconomic status quartile, variance							
1	−5.21	−2.05	−4.08	−6.39	−3.05	−4.12	<.001
2	−2.07	−0.68	−1.63	−3.41	−1.42	−1.60	
3	−0.83	0.82	−0.91	−1.66	−0.26	−0.26	
4	2.39	3.14	1.68	1.33	2.29	2.83	
Missing, No. (%)	676 (11.5)	49 (9.6)	134 (6.9)	176 (9.2)	17 (9.9)	300 (21.7)	
Ethnicity/race							
Non-Hispanic White	509 (8.6)	509 (100)	0	0	0	0	NA
Non-Hispanic Black	1935 (32.8)	0	1935 (100)	0	0	0	
Hispanic	1905 (32.3)	0	0	1905 (100)	0	0	
Asian	171 (2.9)	0	0	0	171 (100)	0	
Other	792 (13.4)	0	0	0	0	792 (57.3)	
Unknown/declined	590 (10.0)	0	0	0	0	590 (42.7)	
Comorbidities, No.							
0	1155 (19.6)	122 (24.0)	266 (13.7)	402 (21.1)	32 (18.7)	333 (24.1)	<.001
1-2	2059 (34.9)	166 (32.6)	743 (38.4)	674 (35.4)	59 (34.5)	417 (30.2)	
>2	1853 (31.4)	147 (28.9)	764 (39.5)	654 (34.3)	44 (25.7)	244 (17.7)	
Missing	835 (14.1)	74 (14.5)	162 (8.4)	175 (9.2)	36 (21.1)	388 (28.1)	NA
Hypertension	2641 (44.7)	218 (42.8)	1042 (53.9)	849 (44.6)	77 (45.0)	455 (32.9)	<.001
BMI							
<18.5	102 (1.7)	13 (2.6)	37 (1.9)	38 (2.0)	3 (1.8)	11 (0.8)	<.001
18.5-35	3888 (65.9)	341 (67.0)	1298 (67.1)	1329 (69.8)	112 (65.5)	808 (58.5)	
>35	1077 (18.2)	81 (15.9)	438 (22.6)	363 (19.1)	20 (11.7)	175 (12.7)	
Missing	835 (14.1)	74 (14.5)	162 (8.4)	175 (9.2)	36 (21.1)	388 (28.1)	NA
Diabetes	1942 (32.9)	124 (24.4)	765 (39.5)	667 (35.0)	64 (37.4)	322 (23.3)	<.001
Cardiovascular disease							
Any	1306 (22.1)	132 (25.9)	549 (28.4)	438 (23.0)	31 (18.1)	156 (11.3)	<.001
Myocardial infarction	366 (6.2)	48 (9.4)	121 (6.3)	146 (7.7)	7 (4.1)	44 (3.2)	<.001
Congestive heart failure	808 (13.7)	88 (17.3)	349 (18.0)	258 (13.5)	20 (11.7)	93 (6.7)	<.001
Peripheral vascular disease	337 (5.7)	32 (6.3)	141 (7.3)	124 (6.5)	8 (4.7)	32 (2.3)	<.001
Cerebrovascular disease	423 (7.2)	34 (6.7)	202 (10.4)	133 (7.0)	10 (5.8)	44 (3.2)	<.001
Chronic pulmonary disease	1250 (21.2)	101 (19.8)	478 (24.7)	474 (24.9)	25 (14.6)	172 (12.4)	<.001
Kidney disease	1175 (19.9)	73 (14.3)	538 (27.8)	384 (20.2)	23 (13.5)	157 (11.4)	<.001
Liver disease	393 (6.7)	31 (6.1)	129 (6.7)	184 (9.7)	8 (4.7)	41 (3.0)	<.001
Cancer	333 (5.6)	31 (6.1)	135 (7.0)	116 (6.1)	4 (2.3)	47 (3.4)	<.001
Dementia	303 (5.1)	41 (8.1)	131 (6.8)	100 (5.2)	4 (2.3)	27 (2.0)	<.001
Peptic ulcer	151 (2.6)	9 (1.8)	61 (3.2)	60 (3.1)	8 (4.7)	13 (0.9)	<.001
Hemiplegia or paraplegia	128 (2.2)	7 (1.4)	60 (3.1)	44 (2.3)	2 (1.2)	15 (1.1)	.001
HIV/AIDS	92 (1.6)	3 (0.6)	37 (1.9)	34 (1.8)	1 (0.6)	17 (1.2)	.13

Abbreviation: BMI, body mass index (calculated as weight in kilograms divided by height in meters squared).

A total of 918 patients (15.5%) died within the study time frame, after allowing for at least a 12-day window between the last day of COVID positivity and final data collection, with death rates broken down by patient demographic characteristics (eTable 1 in the Supplement). Of the 5902 patients, 3231 (54.7%) required inpatient admission, of whom 470 (14.5%) required an ICU stay. The median (interquartile range) age of hospitalized patients was 65 (54-76) years, 2777 of 3136 (88.6%) had at least 1 comorbidity, and 1506 of 3231 (46.6%) had diabetes. Overall, 1096 patients (18.6%) were treated in emergency department only, and 1575 (26.7%) received care in an outpatient setting. With a median (range) time from positive COVID-19 diagnosis to death of 6 (0.2-40) days, death rates were 27.1% for hospitalized patients (876 of 3231), 44.3% for patients admitted to the ICU (208 of 470), 3.0% for those treated in the emergency department only (33 of 1096), and 0.6% for those treated in the outpatient setting (9 of 1575). Of the 3231 patients hospitalized, 1929 (59.7%) were discharged alive, and 426 (13.2%) remained in the hospital on the date of final data acquisition, meaning 5476 (86.8%) had known outcomes. Of patients admitted to the hospital, the median (interquartile range) length of stay was 5 (3-9) days for patients discharged alive and 6 (3-10) days for those who died.

Risk factors associated with survival after COVID-19 infection are shown in eTable 2 in the Supplement for univariable analysis and Table 2 for multivariable analysis. On multivariable analysis, women were at lower risk of death compared with men (hazard ratio [HR], 0.71; 95% CI, 0.62-0.82; *P* < .001), and older age was strongly associated with the risk of death (aged 40-60 years vs ≤40 years: HR, 2.04; 95% CI, 1.34-3.10; aged 61-80 years vs ≤40 years: HR, 5.47; 95% CI, 3.66-8.18; aged >80 years vs ≤40 years: HR, 10.30; 95% CI, 6.75-15.60; *P *for trend < .001). In multivariable analysis, while controlling for age, sex, SES, and comorbidities, patients who identified as Hispanic (HR, 0.77; 95% CI, 0.61-0.98; *P* = .03) and non-Hispanic Black (HR, 0.69; 95% CI, 0.55-0.87; *P* = .002) had somewhat improved survival compared with non-Hispanic White patients. Medical comorbidities found to be risk factors significantly associated with death were morbid obesity (ie, BMI >35), cardiovascular disease, diabetes, kidney disease, and dementia.

**Table 2.** Cox Proportional Hazards Models Showing the Association of Demographic Characteristics and Comorbidities With Overall Survival in Multivariable Analysis for Complete Case and MI Analyses^a^

Factor	Complete case		MI = 20	
	Hazard ratio (95% CI)	*P* value	Hazard ratio (95% CI)	*P* value
Sex				
Men	1 [Reference]	NA	1 [Reference]	NA
Women	0.72 (0.62-0.83)	<.001	0.71 (0.62-0.82)	<.001
Age, y				
≤40	1 [Reference]	NA	1 [Reference]	NA
41-60	1.85 (1.17-2.94)	.009	2.04 (1.34-3.10)	.001
61-80	5.24 (3.37-8.16)	<.001	5.47 (3.66-8.18)	<.001
>80	9.48 (5.98-15.0)	<.001	10.3 (6.75-15.6)	<.001
Socioeconomic status				
Lowest quartile	1 [Reference]	NA	1 [Reference]	NA
Second quartile	1.07 (0.88-1.30)	.51	1.04 (0.86-1.27)	.67
Third quartile	1.20 (0.99-1.45)	.06	1.18 (0.98-1.42)	.09
Highest quartile	1.11 (0.89-1.38)	.36	1.08 (0.88-1.33)	.45
Ethnicity/race				
Non-Hispanic White	1 [Reference]	NA	1 [Reference]	NA
Non-Hispanic Black	0.72 (0.56-0.92)	.009	0.69 (0.55-0.87)	.002
Hispanic	0.79 (0.61-1.01)	.06	0.77 (0.61-0.98)	.03
Asian	1.16 (0.75-1.80)	.49	1.02 (0.67-1.56)	.91
Other	0.75 (0.55-1.01)	.06	0.71 (0.54-0.94)	.02
Unknown or declined	0.91 (0.62-1.33)	.62	0.80 (0.57-1.13)	.21
BMI				
18.5-35	1 [Reference]	NA	1 [Reference]	NA
<18.5	0.94 (0.63-1.40)	.75	0.97 (0.66-1.41)	.86
>35	1.38 (1.15-1.67)	.001	1.42 (1.19-1.70)	<.001
Hypertension	1.02 (0.88-1.19)	.77	1.00 (0.87-1.16)	.97
Cardiovascular disease	1.20 (1.03-1.41)	.02	1.16 (1.00-1.35)	.049
Diabetes	1.21 (1.05-1.40)	.01	1.21 (1.06-1.40)	.007
Cancer	0.88 (0.71-1.09)	.25	0.93 (0.75-1.14)	.47
Liver disease	1.23 (0.99-1.52)	.06	1.16 (0.94-1.44)	.15
Dementia	1.48 (1.21-1.81)	<.001	1.47 (1.21-1.78)	<.001
Chronic pulmonary disease	0.98 (0.84-1.15)	.83	1.01 (0.87-1.17)	.94
Peptic ulcer	0.92 (0.68-1.24)	.58	0.90 (0.67-1.22)	.51
Hemiplegia or paraplegia	1.26 (0.93-1.70)	.14	1.26 (0.93-1.70)	.13
Kidney disease	1.54 (1.32-1.80)	<.001	1.53 (1.32-1.78)	<.001
HIV/AIDS	0.88 (0.51-1.51)	.64	0.93 (0.56-1.54)	.78

Abbreviation: BMI, body mass index (calculated as weight in kilograms divided by height in meters squared); MI, multiple imputation; NA, not applicable.

^a^ Number of comorbidities was not included in the multivariable model because of the strong correlation with the individual comorbidities tabulated.

Death rates among the different ethnic and racial groups were 20.0% for non-Hispanic White patients (102 of 509), 17.2% for non-Hispanic Black patients (333 of 1935), 16.2% for Hispanic patients (309 of 1905), and 17.0% for Asian patients (29 of 171) (*P* = .25). The eFigure in the Supplement shows death rates across age strata among non-Hispanic White, non-Hispanic Black, Hispanic, and Asian patients or patients with other race/ethnicity, stratified by sex and number of medical comorbidities. After stratifying by these risk factors, there were no appreciable differences seen in the death rates between non-Hispanic White patients and patients from other racial/ethnic groups.

Comparing the number of medical comorbidities across ethnic and racial groups, Hispanic and non-Hispanic Black patients had a higher proportion of multiple medical comorbidities (Table 3), and they were also more likely to need inpatient admission (1206 [62.3%] and 1148 [60.2%]) compared with 243 (47.7%) for non-Hispanic White patients (*P* < .001). Differences in management setting by race/ethnicity are further described in eTable 3 in the Supplement. Despite these differences, there were no significant differences in survival between non-Hispanic White, non-Hispanic Black, Hispanic, or Asian patients when examining the full cohort. Stratifying patients by hospitalization status, we found that non-Hispanic White patients requiring hospital or ICU admission had somewhat higher death rates compared with the other ethnic groups (Table 3). A sensitivity analysis excluding the 145 patients (2.5%) who came from chronic nursing facilities (23 [4.5%] non-Hispanic White patients, 49 [2.5%] non-Hispanic Black patients, and 52 [2.7%] Hispanic patients) did not alter the association between race/ethnicity and mortality, with an HR of 0.70 (95% CI, 0.55-0.89) for non-Hispanic Black patients and 0.78 (95% CI, 0.61-1.00) for Hispanic patients compared with non-Hispanic White patients.

**Table 3.** Outcomes and Comorbidities Associated With Ethnicity and Race for Patients With Known Race/Ethnicity

Characteristic	Patients, No./total No. (%)							*P* value
	Non-Hispanic White		Non-Hispanic Black		Hispanic		Asian	
**All patients with known ethnicity identification (n = 4520)**								
Deceased								
Yes	102/509 (20.0)	333/1935 (17.2)		309/1905 (16.2)		29/171 (17.0)		.25
No	407/509 (80.0)	1602/1935 (82.8)		1596/1905 (83.8)		142/171 (83.0)		
Comorbidities, No.								
0	122/435 (28.0)	266/1773 (15.0)		402/1905 (20.8)		32/135 (23.7)		<.001
1-2	166/435 (38.2)	743/1773 (41.9)		674/1905 (34.8)		59/135 (42.7)		
>2	147/435 (33.8)	764/1773 (43.1)		654/1905 (33.8)		44/135 (32.6)		
**Hospitalized patients with known ethnicity identification (n = 2678)**								
Deceased								
Yes	98/243 (40.3)	323/1206 (26.8)		292/1148 (25.4)		27/81 (33.3)		<.001
No	145/243 (59.7)	883/1206 (73.2)		856/1148 (74.6)		54/81 (66.7)		
Comorbidities, No.								
0	18/237 (7.6)	84/1177 (7.1)		156/1116 (14.0)		7/75 (9.3)		<.001
1-2	101/237 (42.6)	445/1177 (37.8)		406/1116 (36.4)		34/75 (45.3)		
>2	118/237 (49.8)	648/1177 (55.1)		554/1116 (49.6)		34/75 (45.3)		
**Patients admitted to the ICU with known ethnicity identification (n = 386)**								
Deceased								
Yes	24/39 (61.5)	74/165 (44.8)		64/160 (40)		9/22 (40.9)		.11
No	15/39 (38.5)	91/165 (55.2)		96/160 (60)		13/22 (59.1)		
Comorbidities, No.								
0	6/39 (15.4)	12/162 (7.4)		25/159 (15.7)		2/22 (9.1)		.12
1-2	15/39 (38.5)	55/162 (34.0)		64/159 (40.3)		10/22 (45.5)		
>2	18/39 (46.2)	95/162 (58.6)		70/159 (44.0)		10/22 (45.5)		

Abbreviation: ICU, intensive care unit.

Risk factors associated with survival, broken down by ethnic and racial grouping, are presented in Table 4 based on multivariable Cox regression. Older age was associated with worse survival in each group except for Asian patients. The association of comorbidities with mortality differed across race/ethnicity categories. While cardiovascular disease was associated with worse survival for non-Hispanic White patients, among non-Hispanic Black patients we found that male sex, a BMI greater than 35, diabetes, kidney disease, and dementia were associated with increased risk of mortality. Hispanic patients with a BMI greater than 35, kidney disease, or hemiplegia/paraplegia had worse survival, while Asian patients with cardiovascular disease, kidney disease, or hemiplegia/paraplegia had worse outcomes.

**Table 4.** Multivariable Cox Models Showing Risk Factors Associated With Overall Survival Using the Multiple Imputation Analysis, Stratified by Ethnic and Racial Group

Risk factor	Multivariable models stratified by ethnic and racial group							
	Non-Hispanic White patients		Non-Hispanic Black patients		Hispanic patients		Asian patients	
	HR (95% CI)	*P* value	HR (95% CI)	*P* value	HR (95% CI)	*P* value	HR (95% CI)	*P* value
Women vs men	0.79 (0.51-1.24)	.31	0.74 (0.59-0.93)	.01	0.81 (0.64-1.03)	.08	1.03 (0.37-2.86)	.95
Age, y								
≤40	1 [Reference]	NA	1 [Reference]	NA	1 [Reference]	NA	1 [Reference]	NA
41-60	0.98 (0.20-4.83)	.98	3.03 (1.29-7.11)	.01	1.68 (0.90-3.13)	.10	1.69 (0.26-11.1)	.58
61-80	2.57 (0.59-11.2)	.21	7.09 (3.08-16.3)	<.001	5.01 (2.76-9.10)	<.001	1.64 (0.24-11.3)	.62
>80	4.88 (1.09-21.8)	.04	12.4 (5.27-29.3)	<.001	11.0 (5.82-20.7)	<.001	1.26 (0.14-11.3)	.84
Socioeconomic status								
Lowest quartile	1 [Reference]	NA	1 [Reference]	NA	1 [Reference]	NA	1 [Reference]	NA
Second quartile	1.29 (0.57-2.92)	.54	1.00 (0.70-1.43)	.98	1.02 (0.76-1.36)	.91	0.61 (0.13-2.88)	.53
Third quartile	1.26 (0.59-2.68)	.55	1.31 (0.96-1.78)	.09	1.10 (0.79-1.55)	.55	0.55 (0.12-2.52)	.44
Highest quartile	1.06 (0.51-2.19)	.87	1.20 (0.84-1.72)	.31	1.29 (0.89-1.87)	.18	0.32 (0.05-2.08)	.23
BMI								
18.5-35	1 [Reference]	NA	1 [Reference]	NA	1 [Reference]	NA	1 [Reference]	NA
<18.5	1.90 (0.74-4.84)	.18	1.01 (0.55-1.86)	.96	0.74 (0.35-1.54)	.42	8.58 (0.72-102)	.09
>35	0.80 (0.41-1.56)	.51	1.42 (1.07-1.87)	.01	1.40 (1.01-1.94)	.04	3.81 (0.97-14.9)	.05
Hypertension	1.55 (0.98-2.47)	.06	1.01 (0.80-1.29)	.91	0.92 (0.72-1.18)	.53	1.50 (0.48-4.64)	.48
Cardiovascular disease	2.30 (1.40-3.79)	.001	1.04 (0.82-1.33)	.74	0.99 (0.76-1.30)	.96	3.12 (1.03-9.45)	.04
Diabetes	1.24 (0.80-1.93)	.33	1.27 (1.01-1.59)	.04	1.24 (0.96-1.59)	.09	0.53 (0.18-1.56)	.25
Cancer	0.60 (0.31-1.15)	.13	1.12 (0.82-1.53)	.48	1.05 (0.72-1.51)	.81	2.56 (0.36-18.1)	.35
Liver disease	0.61 (0.24-1.55)	.30	1.01 (0.71-1.45)	.94	1.33 (0.97-1.84)	.08	1.66 (0.24-11.4)	.61
Dementia	1.21 (0.69-2.13)	.51	1.62 (1.19-2.21)	.002	1.38 (0.98-1.94)	.061	4.30 (0.68-27.0)	.12
Chronic pulmonary disease	0.84 (0.52-1.37)	.48	1.14 (0.89-1.47)	.30	0.96 (0.74-1.23)	.72	0.47 (0.12-1.86)	.28
Peptic ulcer	2.25 (0.76-6.64)	.14	0.70 (0.41-1.17)	.17	1.09 (0.68-1.74)	.72	0.75 (0.14-4.11)	.74
Hemiplegia or paraplegia	1.55 (0.50-4.84)	.45	1.00 (0.63-1.61)	.99	1.96 (1.20-3.22)	.007	15.8 (2.01-124)	.009
Kidney disease	1.29 (0.82-2.02)	.27	1.74 (1.36-2.23)	<.001	1.31 (1.01-1.70)	.04	4.27 (1.40-13.1)	.01
HIV/AIDS	NA^a^	NA	1.14 (0.55-2.39)	.72	0.77 (0.31-1.92)	.58	NA^b^	NA

Abbreviations: BMI, body mass index (calculated as weight in kilograms divided by height in meters squared); HR, hazard ratio; NA, not applicable.

^a^ Models did not converge for the following reasons: only 3 of 509 patients had HIV/AIDS and none died.

^b^ Models did not converge for the following reasons: only 1 of 171 patients had HIV/AIDS and did not die.

## Discussion

In this sample of 5902 ethnically diverse patients with COVID-19 who were treated at a single large academic medical center in the Bronx, New York, non-Hispanic Black and Hispanic patients, despite presenting with higher numbers of comorbidities and being more likely to require inpatient hospitalization, had outcomes at least as good as, and maybe even marginally superior to, their non-Hispanic White counterparts after controlling for age, sex, and comorbidities. This finding that Black and Hispanic patients did not experience worse outcomes compared with their White counterparts when presenting for care at the same urban medical center is important for understanding the observed population differences in mortality by race/ethnicity reported elsewhere. Do the findings reported here conflict with observations that the population-based mortality rates for COVID-19 are higher for Black and Hispanic populations than for White populations?^9^ Can we reconcile the findings with reports of elevated case fatality rates for Black and Hispanic patients relative to White patients?

Our findings that non-Hispanic Black and Hispanic patients with COVID-19 had mortality rates at least as good as their White counterparts does not speak directly to the elevated burden of this disease among minority populations. To the extent that more non-Hispanic Black and Hispanic individuals contracted the infection during the period under study, the observed population-based mortality rates would be elevated relative to white populations even if non-Hispanic Black and Hispanic patients with infection had similar outcomes as White patients.

Differences between our estimates of equality among racial and ethnic groups in case fatality rates for COVID-19 relative to other estimates may derive from differential testing for COVID-19 in our sample compared with the testing patterns in other samples reporting case fatality rates. If non-Hispanic Black and Hispanic patients not receiving care in a well-integrated health care delivery system are only tested when they show severe symptoms compared with their White counterparts, whose access to care permits a broader testing strategy, estimates of the case fatality rate for COVID-19 may be falsely elevated compared with the estimates that would be reported if testing regimens were equivalent across race/ethnicity classifications.

Are the characteristics of our sample population comparable with those of other published series? Similar to other reports, we found that older age and multiple comorbidities, including obesity, cardiovascular disease, diabetes, kidney disease, and dementia, were associated with poor outcomes from COVID-19, and this remained consistent across ethnic and racial groups.

An April 2020 article^10^ reported on differences in COVID-19 testing, hospitalizations, and outcomes among the different New York City boroughs, showing higher rates of hospitalization and death in the Bronx. The authors reported only unadjusted rates for these metrics. Contrasting this to our study, the importance of controlling for risk factors, such as age and comorbidities, to accurately estimate outcome differences in ethnically and racially diverse populations, such as that of New York City, becomes clear.

Comparing the demographic characteristics of hospitalized patients in our sample with other recent reports from the United States, we find important similarities between patients hospitalized at Montefiore and those from other institutions, with 88.6% of our hospitalized patients presenting with medical comorbidities compared with 94% patients hospitalized within the Northwell Health System in New York.^4^ In contrast, our hospitalized patients were slightly older, with a median age of 65, compared with 63 and 61 for patients hospitalized at Northwell and at Kaiser Permanente in Northern California,^3^ respectively. Further differences included a higher proportion of patients at Montefiore presenting with diabetes (46.6% vs 34% and 31%, respectively). The older age and higher rates of diabetes seen in our patients might account for the higher mortality in hospitalized patients.

A July 2020 report^11^ from another large health care system in California showed that Black patients were more likely to be hospitalized with COVID-19, which agrees with the higher rates of inpatient admission seen among non-Hispanic Black patients in our cohort. A major difference between this cohort and our cohort is that Montefiore serves a large proportion of patients who belong to ethnic and racial minority groups, and despite a larger proportion of these patients requiring inpatient admission, they did not have worse outcomes in terms of mortality.

The stratified design of this study permitted a detailed presentation of comorbidities by race/ethnicity and fully interactive analytic approach that highlighted differences in outcomes among ethnic and racial groups while controlling for all comorbidities in addition to age and sex. Although non-Hispanic White patients were slightly older and had fewer comorbidities, stratified multivariate analysis indicated that outcomes from COVID-19 were not notably different compared with patients from other racial and ethnic groups. Another strength of this analysis is that only a small number of hospitalized patients (13.2%) were still admitted by end of data collection, with 86.8% of patients having known outcome disposition.

### Limitations

This study has limitations, including its retrospective nature, with data availability limited to the electronic medical records at our institution. This study also did not address differences in the rates of COVID-19 positivity or access to health care among the different ethnic and racial groups, both of which could affect cumulative mortality numbers. Importantly, the case fatality rates presented here are conditional on patients having tested positive for COVID-19. If the likelihood of being tested varies systematically across race/ethnicity and SES or if potential differences in false-negative rates among different groups exist, the ratios of case fatality rates across race/ethnicity categories would be affected. Furthermore, approximately 4.5% of non-Hispanic White patients in this cohort were transferred from chronic nursing facilities; thus, these patients may not be representative of the general non-Hispanic White population in a variety of unobservable characteristics that could not be controlled for. However, excluding patients coming from nursing homes did not change the association between race/ethnicity and mortality.

## Conclusions

In this study, Black and Hispanic patients who tested positive for COVID-19 and received care at a large academic health system in a densely populated urban environment experienced similar outcomes as their White counterparts when controlling for age, sex, and comorbid conditions at presentation. These findings may provide some reassurance that access to the services available in comprehensive health care environments may attenuate, if not eliminate, racial/ethnic differentials in COVID-19 mortality rates.

## Notes

Supplement. **eFigure. **Death Rates Across Age Strata Among Non-Hispanic White Patients, Non-Hispanic Black Patients, Hispanic Patients, and Asian Patients or Patients with Other Race/Ethnicity, Stratified by Sex and Number of Medical Comorbidities **eTable 1. **Death Rates By Patient Demographic Characteristics **eTable 2.** Cox Proportional Hazards Models for the Association of Demographic Characteristics and Comorbidities With Overall Survival in Univariable Analysis for Complete Case and Multiple Imputation Analyses **eTable 3.** Management Setting Shown by Race and Ethnicity and the Association Between Socioeconomic Status and Number of Comorbidities
