# Supplementary material for: Association of Race and Ethnicity With Comorbidities and Survival Among Patients With COVID-19 at an Urban Medical Center in New York
Source: JAMA Netw Open. 2020 Sep 25;3(9):e2019795. doi: 10.1001/jamanetworkopen.2020.19795 (PMC7519416; doi:10.1001/jamanetworkopen.2020.19795)
Supplement: Supplement. — eFigure. Death Rates Across Age Strata Among Non-Hispanic White Patients, Non-Hispanic Black Patients, Hispanic Patients, and Asian Patients or Patients with Other Race/Ethnicity, Stratified by Sex and Number of Medical Comorbidities eTable 1. Death Rates By Patient Demographic Characteristics eTable 2. Cox Proportional Hazards Models for the Association of Demographic Characteristics and Comorbidities With Overall Survival in Univariable Analysis for Complete Case and Multiple Imputation Analyses eTable 3. Management Setting Shown by Race and Ethnicity and the Association Between Socioeconomic Status and Number of Comorbidities [file jamanetwopen-e2019795-s001.pdf]

Supplementary Online Content

Kabarriti R, Brodin NP, Maron MI, et al. Association of race and ethnicity with comorbidities and survival among patients with COVID-19 at an urban medical center in New York. *JAMA Netw Open*. 2020;3(9):e2019795. doi:10.1001/jamanetworkopen.2020.19795

eFigure. Death Rates Across Age Strata Among Non-Hispanic White Patients, Non-Hispanic Black Patients, Hispanic Patients, and Asian Patients or Patients with Other Race/Ethnicity, Stratified by Sex and Number of Medical Comorbidities
eTable 1. Death Rates By Patient Demographic Characteristics
eTable 2. Cox Proportional Hazards Models for the Association of Demographic Characteristics and Comorbidities With Overall Survival in Univariable Analysis for Complete Case and Multiple Imputation Analyses
eTable 3. Management Setting Shown by Race and Ethnicity and the Association Between Socioeconomic Status and Number of Comorbidities

This supplementary material has been provided by the authors to give readers additional information about their work.

© 2020 Kabarriti R et al. *JAMA Network Open*.

eFigure. Death Rates Across Age Strata Among Non-Hispanic White Patients, Non-Hispanic Black Patients, Hispanic Patients, and Asian Patients or Patients with Other Race/Ethnicity, Stratified by Sex and Number of Medical Comorbidities

Asian patients or patients with other race/ethnicity were grouped together due to the relatively low number of Asian patients in our cohort.

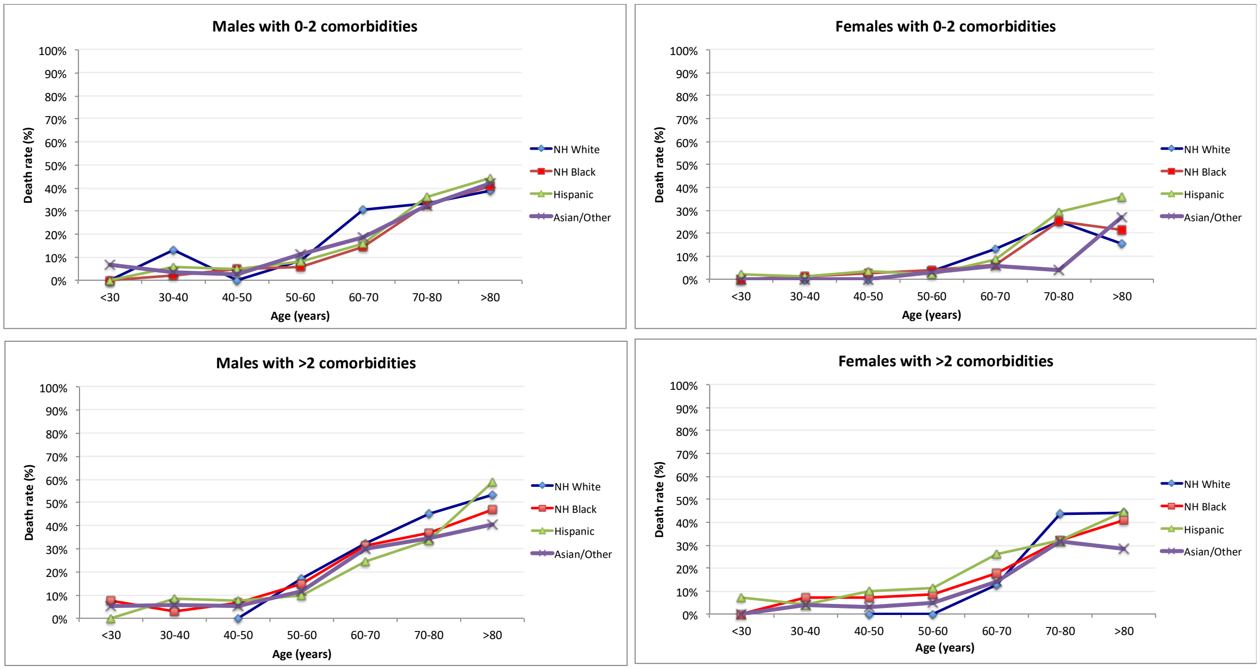

© 2020 Kabarriti R et al. *JAMA Network Open*.

eTable 1. Death Rates By Patient Demographic Characteristics

	Nr of deaths (% death rate)
Sex	
Male	522 (18.9%)
Female	396 (12.7%)
Age	
≤40	27 (2.1%)
41-60 years	133 (6.6%)
61-80 years	507 (25.3%)
>80 years	251 (40.5%)
Socioeconomic status	
Lowest quartile	220 (14.9%)
2nd quartile	206 (13.7%)
3rd quartile	280 (19.4%)
Highest quartile	212 (14.3%)
Ethnicity/Race	
Non-Hispanic White	102 (20.0%)
Non-Hispanic Black	333 (17.2%)
Hispanic	309 (16.2%)
Asian	29 (17.0%)
Other	95 (12.0%)
Nr of comorbidities	
0	56 (3.1%)
1-2	276 (12.3%)
>2	586 (31.3%)
BMI	
18.5-35	719 (15.3%)
<18.5	29 (28.4%)
>35	170 (15.3%)
Hypertension	
No	319 (9.8%)
Yes	599 (22.7%)
Cardiovascular disease	
No	468 (10.2%)
Yes	450 (34.5%)
Diabetes mellitus	
No	408 (10.3%)
Yes	510 (26.3%)
Cancer	
No	809 (14.5%)
Yes	109 (32.7%)
Liver disease	
No	805 (14.6%)
Yes	113 (28.8%)
Dementia	
No	776 (13.9%)
Yes	142 (46.9%)
Chronic pulmonary disease	
No	640 (13.8%)
Yes	278 (22.2%)
Peptic ulcer	
No	869 (15.1%)
Yes	49 (32.5%)
Hemiplegia/Paraplegia	
No	868 (15.0%)
Yes	50 (39.1%)
Renal disease	
No	455 (9.6%)
Yes	463 (39.4%)
HIV/AIDS	
No	902 (15.5%)
Yes	16 (17.4%)

© 2020 Kabarriti R et al. *JAMA Network Open*.

eTable 2. Cox Proportional Hazards Models for the Association of Demographic Characteristics and Comorbidities With Overall Survival in Univariable Analysis for Complete Case and Multiple Imputation Analyses

	Complete case		MI = 20	
	**Hazard Ratio (95% CI)**	**P-value**	**Hazard Ratio (95% CI)**	**P-value**
Sex				
Male	(ref)		(ref)	
Female	0.67 (0.59, 0.76)	<0.001	0.67 (0.59, 0.76)	<0.001
Age				
≤40	(ref)		(ref)	
41-60 years	2.42 (1.60, 3.66)	<0.001	2.42 (1.60, 3.66)	<0.001
61-80 years	7.63 (5.18, 11.2)	<0.001	7.63 (5.18, 11.2)	<0.001
>80 years	14.9 (9.98, 22.1)	<0.001	14.9 (9.98, 22.1)	<0.001
Socioeconomic status				
Lowest quartile	(ref)		(ref)	
2nd quartile	1.11 (0.92, 1.35)	0.27	1.11 (0.91, 1.34)	0.31
3rd quartile	1.36 (1.13, 1.63)	0.001	1.34 (1.12, 1.61)	0.002
Highest quartile	1.13 (0.92, 1.38)	0.23	1.13 (0.92, 1.38)	0.24
Ethnicity/Race				
Non-Hispanic White	(ref)		(ref)	
Non-Hispanic Black	0.68 (0.54, 0.85)	0.001	0.68 (0.54, 0.85)	0.001
Hispanic	0.66 (0.52, 0.82)	<0.001	0.66 (0.52, 0.82)	<0.001
Asian	0.79 (0.53, 1.20)	0.28	0.79 (0.53, 1.20)	0.28
Other	0.57 (0.43, 0.76)	<0.001	0.57 (0.43, 0.76)	<0.001
Unknown/declined	0.56 (0.40, 0.78)	0.001	0.56 (0.40, 0.78)	0.001
Nr of comorbidities				
0	(ref)		(ref)	
1-2	1.66 (1.18, 2.34)	0.004	1.66 (1.18, 2.34)	0.004
>2	3.52 (2.54, 4.89)	<0.001	3.52 (2.54, 4.89)	<0.001
BMI				
18.5-35	(ref)		(ref)	
<18.5	1.37 (0.95, 1.99)	0.094	1.37 (0.94, 1.99)	0.096
>35	0.89 (0.75, 1.05)	0.17	0.88 (0.74, 1.04)	0.13
Hypertension	1.58 (1.38, 1.81)	<0.001	1.58 (1.38, 1.81)	<0.001
Cardiovascular disease	2.35 (2.06, 2.67)	<0.001	2.35 (2.06, 2.67)	<0.001
Diabetes mellitus	1.82 (1.60, 2.07)	<0.001	1.82 (1.60, 2.07)	<0.001
Cancer	1.56 (1.28, 1.91)	<0.001	1.56 (1.28, 1.91)	<0.001
Liver disease	1.30 (1.07, 1.58)	0.009	1.30 (1.07, 1.58)	0.009
Dementia	3.06 (2.56, 3.66)	<0.001	3.06 (2.56, 3.66)	<0.001
Chronic pulmonary disease	1.16 (1.01, 1.34)	0.035	1.16 (1.01, 1.34)	0.035
Peptic ulcer	1.56 (1.17, 2.08)	0.002	1.56 (1.17, 2.08)	0.002
Hemiplegia/Paraplegia	1.90 (1.43, 2.52)	<0.001	1.90 (1.43, 2.52)	<0.001

© 2020 Kabarriti R et al. *JAMA Network Open*.

Renal disease	2.68 (2.35, 3.05)	<0.001	2.68 (2.35, 3.05)	<0.001
HIV/AIDS	0.88 (0.53, 1.44)	0.60	0.88 (0.53, 1.44)	0.60

© 2020 Kabarriti R et al. *JAMA Network Open*.

eTable 3. Management Setting Shown by Race and Ethnicity and the Association Between Socioeconomic Status and Number of Comorbidities

	**Management setting**		
	Outpatient	Admitted to hospital	Sent to the ED but not admitted
**Ethnicity/Race**			
Non-Hispanic White	202 (40%)	243 (48%)	64 (12%)
Non-Hispanic Black	411 (21%)	1206 (62%)	318 (16%)
Hispanic	276 (14%)	1148 (60%)	481 (25%)
Asian	56 (33%)	81 (47%)	34 (20%)
Other	306 (39%)	362 (46%)	124 (16%)
P-value	<0.001		

	**Nr of comorbidities**			
	0	1-2	>2	
	Median SES (inter-quartile range)			P-value
**Ethnicity/Race**				
Non-Hispanic White	0.45 (-1.33, 1.90)	-0.70 (-1.98, 0.57)	-1.10 (-2.39, -0.17)	<0.001
Non-Hispanic Black	-1.38 (-3.47, -0.76)	-1.68 (-4.08, -0.81)	-1.63 (-4.80, -1.04)	0.11
Hispanic	-3.61 (-6.18, -1.91)	-3.19 (-6.39, -1.47)	-3.54 (-6.46, -1.56)	0.28
Asian	-1.27 (-2.13, 0.50)	-2.27 (-3.95, -0.33)	-1.94 (-3.64, -1.00)	0.096
Oher	-1.59 (-4.16, 0.21)	-1.79 (-3.36, -0.58)	-2.02 (-5.08, -0.96)	0.033

© 2020 Kabarriti R et al. *JAMA Network Open*.
